# Innovative Method for Reliable Measurement of PEM Water Electrolyzer Component Resistances

**DOI:** 10.1002/smtd.202401842

**Published:** 2025-01-24

**Authors:** Nikolai Utsch, Florian Berg, Fabian Scheepers, Sebastian Holtwerth, Meital Shviro, Werner Lehnert, Anna K. Mechler

**Affiliations:** ^1^ Forschungszentrum Juelich GmbH Institute of Energy Technologies IET‐4 Electrochemical Process Engineering 52425 Juelich Germany; ^2^ RWTH Aachen University Faculty of Mechanical Engineering 52056 Aachen Germany; ^3^ Chemistry and Nanoscience Center National Renewable Energy Laboratory (NREL) Golden CO 80401 USA; ^4^ RWTH Aachen University Faculty of Mechanical Engineering Modeling in Electrochemical Process Engineering 52056 Aachen Germany; ^5^ RWTH Aachen University Electrochemical Reaction Engineering (AVT.ERT) Forckenbeckstr. 51 52074 Aachen Germany; ^6^ Jülich Aachen Research Alliance JARA‐Energy 52066 Aachen Germany

**Keywords:** catalyst layers, image processing, in‐plane electrical resistivities, polymer electrolyte membrane (PEM) water electrolyzers, porous electrodes, printed circuit boards (PCBs), sheet resistances

## Abstract

Understanding the sheet resistance of porous electrodes is essential for improving the performance of polymer electrolyte membrane (PEM) water electrolyzers and related technologies. Despite its importance, existing methods often fail to provide reliable and comprehensive data, especially for porous materials with complex morphologies and non‐uniform thicknesses. This study introduces a robust and straightforward method for determining the sheet resistance of porous electrodes using a novel probe concept based on industrial printed circuit board (PCB) technology. This probe measures resistance across ten distances, ranging from 250 µm to 2500 µm, enabling local mapping of resistance. The study focuses on the sheet resistance of key components in PEM water electrolyzers, including the gas diffusion layer (GDL), porous transport layer (PTL), and catalyst layers deposited on a membrane. Additionally, an image‐processing‐based method is presented to obtain the thickness distribution of the studied catalyst layers, facilitating a detailed analysis of the electrical in‐plane resistivity with thickness variations. Overall, this methodology has the potential to expedite material integration and bridge the gap between electrode engineering and single‐cell testing, thereby advancing the development of PEM water electrolyzers.

## Graphical abstract

Accurate sheet resistance measurements are essential for optimizing porous electrode design in polymer electrolyte membrane water electrolyzers. The authors present a reliable method using a novel probe concept based on industrial printed circuit board technology. Furthermore, the authors use image processing to extract the thickness distribution of the catalyst layer, which is discussed in relation to the in‐plane electrical resistivity.

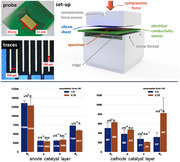

## 1 Introduction

Achieving a sustainably driven energy market is crucially connected to quick and successful progress in the field of energy conversion devices. Proton exchange membrane (PEM) water electrolysis is a technology that features attractive hydrogen production rates combined with high levels of efficiency, aiding in the effective utilization of renewable energy. However, costly components, sluggish anode kinetics, and the required usage of scarce metals continue to delay the comprehensive market penetration of PEM water electrolysis technology.^[^ ^1^, ^2^ ^]^ Although research on single components has gained momentum in recent years, gaps in understanding their system integration continue to hamper progress from the manufacturer's perspective.^[^ ^3^, ^4^, ^5^ ^]^

The main components of an electrolyzer cell are the porous transport layer (PTL), gas diffusion layer (GDL), and membrane, as well as the anode and cathode catalyst layers (CCLs).^[^ ^6^ ^]^ Research has tended to focus on the catalyst itself instead of the catalyst layer or catalyst integration.^[^ ^3^, ^4^, ^7^ ^]^ It is often overlooked that the production of the catalyst layer leads to its own intrinsic properties, entangled in the interaction with specific substrates (e.g., membranes, PTLs), and their interfaces.^[^ ^8^, ^9^, ^10^ ^]^ The manufacturing method and required dispersion determines the catalyst structure initially, although performance also depends on the utilized metal loading with various catalyst–ionomer interactions, affected by its homogenous or in‐homogenous morphology possessing different porosity and thickness distributions.^[^ ^11^, ^12^, ^13^, ^14^ ^]^ Accessing these properties by means of single‐cell testing is also not a trivial task, as many factors, such as cell compression,^[^ ^15^, ^16^, ^17^ ^]^ various interfaces,^[^ ^18^, ^19^, ^20^ ^]^ and electrochemical testing protocols,^[^ ^21^, ^22^ ^]^ affect the results and blur the distinct interpretation of the data. Thus, optimizing and evaluating different catalyst layer designs in membrane electrode assemblies (MEAs) remains challenging, requiring methods to bridge the complexity between catalyst screening, catalyst layer design, and single‐cell testing results.^[^ ^18^, ^23^ ^]^ Studying the electrical resistivity of the catalyst layer helps to explain effects that emerge during single‐cell testing while offering another brick to describe the catalyst layer microstructure and its electronic nature.^[^ ^11^, ^24^ ^]^ Moreover, the interfacial contact resistance between PTLs and catalyst layers requires special attention.^[^ ^25^, ^26^, ^27^ ^]^

Various methods are described in the literature for determining the sheet resistance or in‐plane electrical resistivity of thin films, catalyst layers, or multilayer systems.^[^ ^28^, ^29^, ^30^, ^31^ ^]^ Ahadi et al. introduced the transfer length method to the study of production parameters,^[^ ^11^ ^]^ whereas other studies used this method to analyze Nafion/PEDOT‐PSS mixtures,^[^ ^32^ ^]^ iridium catalyst compositions,^[^ ^33^ ^]^ or carbon graphitization temperature.^[^ ^23^ ^]^ Furthermore, Nafion swelling and its impact on the in‐plane electrical resistivity studied by the van‐der‐Pauw and four‐electrode methods revealed that the expanded Nafion distorts the layer conductivity by separating the catalyst particles.^[^ ^24^, ^34^ ^]^ Insights into the contact resistance were mostly provided by impedance data recorded during single‐cell testing and correlated to performance losses^[^ ^35^, ^36^ ^]^ or degradation.^[^ ^37^, ^38^ ^]^ For PEM electrolyzers, the in‐plane resistivity of the catalyst layers is a critical parameter. While reducing iridium loading is essential due to its scarcity, it results in higher in‐plane resistivity. This increase results in higher voltage losses, which ultimately reduces the performance and efficiency of the electrolyzer.^[^ ^35^ ^]^ From an electrode engineering perspective, these methods are suitable, but a rapid iterative method would be advantageous for electrode improvement. Most of the methods used at present are custom‐made set‐ups with limited possibilities to be flexibly applied as a standardized method for all PEM water electrolyzer components (PTLs, GDLs, and MEAs). The proposed experimental procedures regarding the transfer length method suffer from maintaining the current collector distances between measurements, which crucially affects contact resistivity.^[^ ^39^ ^]^ Displacement errors of the current collectors would directly affect the measurement result. Accessing the resistivity using the four‐point probe method or van‐der‐Pauw method requires uniformity regarding resistivity and thickness, together with the absence of surface holes.^[^ ^28^ ^]^ In the case of the traditional four‐point method, control of the contact pressure and electrode‐depth remains challenging, whereas there is a lack of spatial resolution at a defined scale.^[^ ^40^ ^]^ Instead, the presented four‐line probe adaptation of the commonly‐used four‐point method allows pressure control, aiding in the adjustment of the contact between specimen and probe while being a robust method with an appropriate signal‐to‐noise ratio.^[^ ^40^, ^41^ ^]^ These advantages of the four‐line probe method over traditional methods make it more suitable for characterizing porous electrodes such as catalyst layers. Moreover, another point often neglected by the presented methods is the accurate determination of the thickness distribution, suggesting a uniform layer, which rather describes the ideal than the real case.

In this work, we present a four‐line probe with ten different spatial resolutions ranging from 250 to 2500 µm, pressure control, and the possibility of monitoring the local electrical resistances multiple times within a total area of 11 mm x 20 mm. The probe was factory‐made and validated by using a commercially available reference specimen with defined sheet resistivity. We extended the method by determining the in‐plane electrical resistivity for titanium‐PTL, GDL, and catalyst layers. For enhanced accuracy regarding the catalyst layer in‐plane electrical resistivity, we used open‐source image‐processing techniques to obtain the thickness distribution from catalyst layers coated onto a membrane.

## 2 Measurement Method & Probe Design

**Figure 1:**
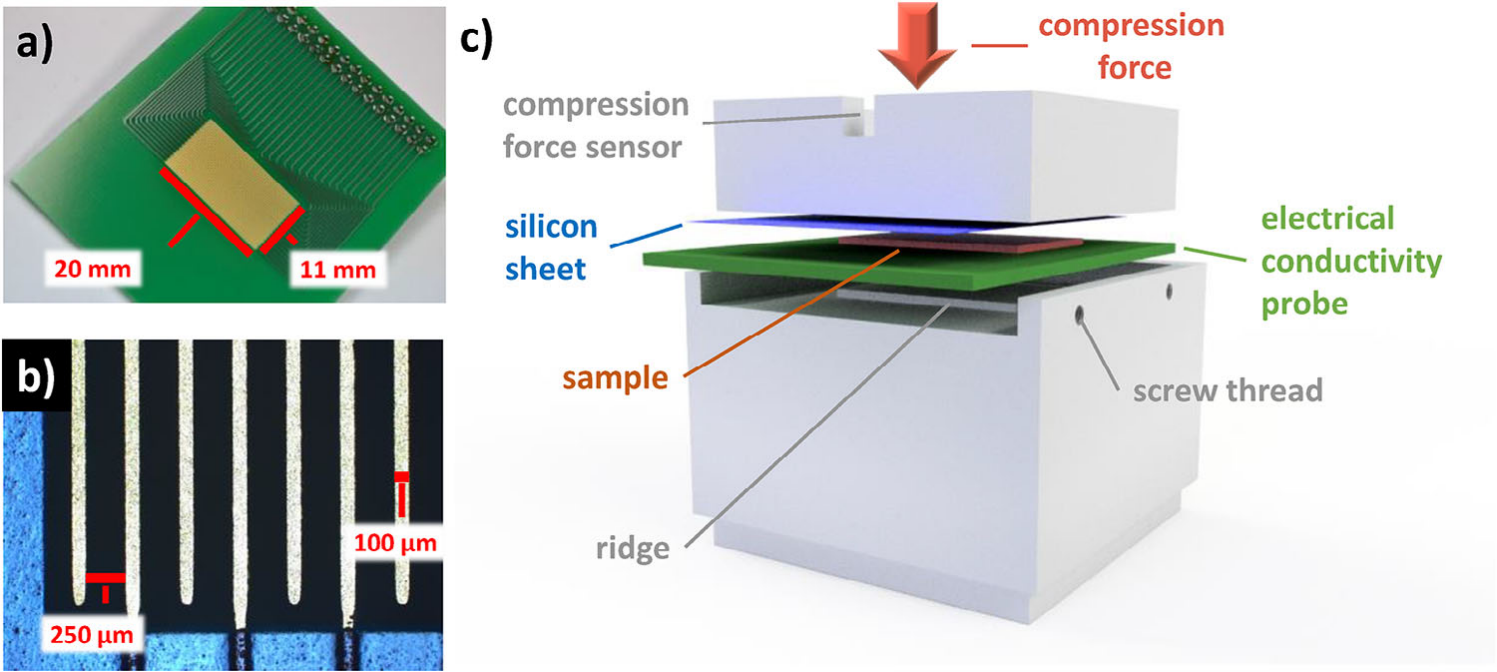
Experimental set up to determine the in‐plane electrical resistance of the PEM water electrolyzer components. The factory‐made probe (a) possessed 32 traces. Each of the traces is 20 mm long and the area of the measuring field was 2.2 cm^2^. A close‐up image obtained via optical microscopy (b) showed the blue solder resist at the border of the measuring field. The traces were 100 µm in width and 250 µm distance apart from one another. To determine the resistance, the specimen was sandwiched between the probe, a silicon sheet, and two aluminum blocks (c).

An accurate measurement of the resistance requires a proper and reliable way to contact the specimen with the measuring field of the probe while controlling the pressure distribution. The probe is presented in **Figure** **1**a with red lines marking the width and length of the measuring field. The image in Figure 1b, taken by an optical microscope (Axio Imager.M1 m, Zeiss), shows the golden traces of the measurement field, and the blue‐colored solder resist. We developed an in‐house manufactured set up to control the contact between the probe and specimen, which is presented in Figure 1c. The set up consists of a stainless steel frame with a screw perpendicular to the middle of the frame bottom, which was used to adjust the compression force. The specimen and probe were sandwiched between two aluminum metal blocks machined with a tolerance of 5 µm. The first block possesses a rectangular cavity in which the probe can be placed and fixated by using two screws on the side. This fixation facilitates the placement of the specimen, which is placed upside down on the measuring field and covered with an insulating silicon sheet with a thickness of 462 ± 4 µm, which ensures a uniform pressure distribution. The second block placed on top of the silicon sheet prevents misalignment while having contact with the protruding border of the cavity. Adjustment of the compression was performed by tightening the screw while monitoring the force using a compression force sensor (K‐14, Lorenz Messtechnik GmbH) and hand‐held measuring amplifier (GM77, Lorenz Messtechnik GmbH). The ridge of the bottom block distributes the compression force uniformly onto the measuring field.

The presented experimental set up was placed in a climate chamber (WKL64/40, Weiss Technik GmbH) to maintain a 25 °C temperature and 25% relative humidity. The specimens were stored inside the climate chamber prior to usage. Before the start of the measurement, the compression force was adjusted 30 min in advance so that the monitored compression force would be constant. The materials tested in this work were measured at a compression force of 1.00 kN first and then at 2.25 kN. Controlling of the potential/current was carried out with a battery cycler (BCS‐815, BioLogic).

### 2.1 Fabrication of the Probe

To facilitate the reproducibility of the electrical resistance measurement, we conceptualized the probe architecture, but ordered it from Würth Elektronik GmbH & Co. KG, a specialist in printed circuit boards (PCBs). Using PCBs as probes has the advantage that the probe production fulfils the IPC A 600 Class 2, and that further customization is possible while maintaining the quality. The material of the rigid board is a 1.55 mm‐thick TG150 FR4, possessing a surface resistivity and volume resistivity ranging above 10^3^ MΩ and 10^3^ MΩ cm, respectively. The traces were made of copper with a final thickness of 35 µm according to the generic standard on printed board design (IPC 2221A). Additionally, the traces were coated with chemical nickel–gold to obtain a nickel thickness of 4 to 7 µm and 0.05 to 0.1 µm gold surface finishing. A solder resist (ELPEMER SD 2467 SM‐DG) was used to insulate the traces, while the area for contacting the specimen remained uncoated. The solder resist prevents short circuits during the soldering process and protects against wetting while ensuring that the specimen only has electrical contact with the measurement field. The measurement field with dimensions of 11 mm x 20 mm (Figure 1a) consists of 32 parallel traces with widths of 100 µm, a length of 20 mm, 250 µm apart from each other, and a thickness of ≈42 µm. Each trace was connected to a drilled hole with an annular ring around it to solder a connection onto the probe. We then soldered a precision socket strip (2 rows, 32 pins) onto the probe and connected this via ribbon cables to soldered 2 mm banana sockets on the other side of the cable to connect a potentiostat. Hence, each banana socket refers to a specific trace, allowing us to measure the resistance while using different equidistant configurations, made accessible by simply and quickly re‐plugging the connections. The price per probe was less than €20 through ordering 15 in total. These low costs make the probe accessible to any laboratory bench.

### 2.2 Measurement Method

**Figure 2:**
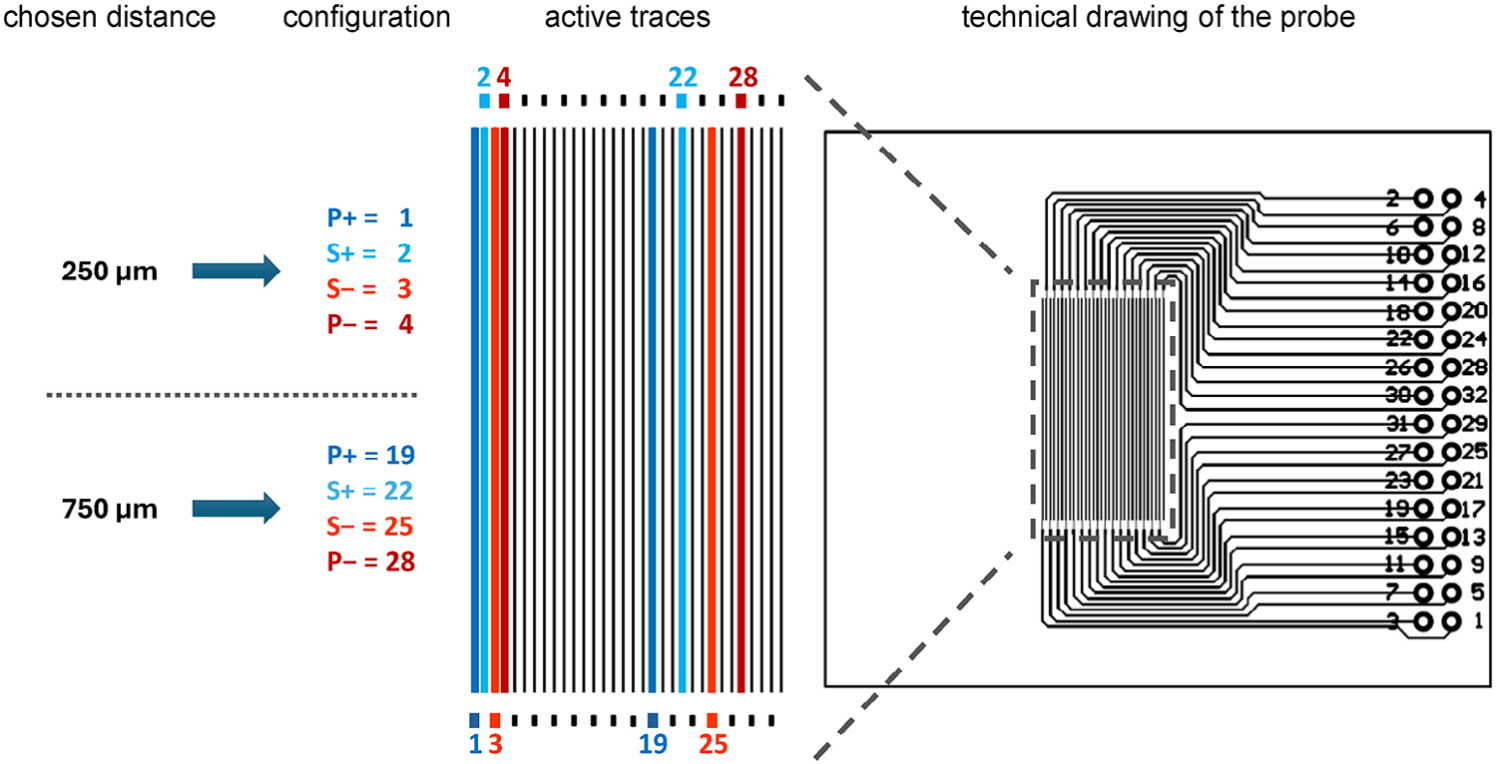
Exemplary selection of the distance of interest obtained by connecting a particular combination of traces. The selected distances were 250 and 750 µm. Both could be measured by a variety of combinations, as shown in Figure S1 (Supporting Information). The outer tracks are labeled P+ and P‐, whereas the inner ones are labeled S+ and S‐. The 250 µm can be measured by using the traces^[^ ^1^, ^2^, ^3^, ^4^ ^]^ labeled in the technical drawing of the probe. A possible configuration for measuring the 750 µm distance.^[^ ^19^, ^22^, ^25^, ^28^ ^]^

The resistance was measured by applying voltage or current on the two outer traces (P+, P‐) while sensing the response using the two inner traces (S+, S‐). As an example, **Figure** **2** shows the connected traces for the 250 and 750 µm as typically chosen distances. According to the corresponding configuration, the active traces were connected to the potentiostat to determine the resistance. The technical drawing of the probe shows the numbering of the 32 traces to choose the configuration of interest. According to Ohm's law, a truly Ohmic resistance is a linear function of the voltage and current. Hence, to ensure the linearity of the measurement, a potential scan was performed, which increases the potential linearly in time. The potential window set for all materials tested ranged from 0 mV to 5 mV at a scan rate of 0.5 mV s^−1^. By plotting the voltage against the measured current and performing a linear regression, the resistance was obtained from the slope. For a four‐line measurement, only four traces were needed, but due to the design of the probe there were 155 configurations for performing the measurement with a total of ten different equidistant distances (*d*) of the contacted traces, ranging from 250 to 2500 µm. Using a distance of 250 µm allows us to measure 29 equidistant configurations, whereas the next larger distance (+ 250 µm) reduces the number of possible configurations by three. Thus, the distance of 2500 µm can only be measured by two configurations. A detailed overview of the configurations related to the distance of interest can be found in Figure S1 (Supporting Information). Especially for catalyst layers, we expect a high locality of the resistances to be obtained by variations in thickness, loading and composition. Thus, for each distance (*d*) we measured three different configurations that were randomly chosen across the measuring field and averaged the obtained resistances per distance.

Two parameters commonly used to characterize thin or thick layers are the in‐plane electrical conductivity (*σ*) and in‐plane electrical resistivity (*ρ*), which are proportional to the specimen thickness (*t*). However, the traditional four‐point methods assume a uniform thickness, which is challenging to maintain for porous metal–polymer electrodes deposited onto a membrane, whose spatial extent is sensitive to relative humidity. Therefore, sheet resistance (*R_s_*) is an interesting alternative as it can be determined unaffected by the uncertainty of a non‐uniform thickness, as depicted in Equation (1):

(1) $$R_{s}=\frac{\rho }{t}$$

Accordingly, measuring only one equidistant space between the traces remains prone to error, as it does not use the beneficial averaging effects to reduce noise. The 20 mm length of the traces ensures sufficient contact between the probe and specimen. Thus, in practice, we determine the resistance (*R*) as described before and plot the averaged values against the distance (*d*) between the selected traces. The relationship of Equation 2 is used to obtain the sheet resistance by means of linear regression analysis. Equation 2 states that the slope is equal to the sheet resistance of the material divided by the length of the traces (*w*). Any additional resistance in the circuit can arise from a variety of sources, such as contact resistance between the probe and specimen, cable resistance, and joint resistances. The collective impact of these additional resistances is represented by the y‐axis intercept (*R_0_*):

(2) $$R\;=R_{s}\frac{d}{w}+R_{0}$$

The y‐intercept represents a series of resistors that were invariably measured and consisted, among others, of the cable resistances, contact resistances, and variation in the solder joints. However, the elements of the resistor series were kept constant. Therefore, a change in the y‐intercept corresponds to an altered contact resistance (*R_c_*) between the probe and specimen.

Another strength of the measurement technique is the possibility of monitoring the spatially‐resolved resistances across the specimen (1D mapping). For such a measurement, all configurations at, e.g., 250 µm are tracked individually, i.e.,^[^ ^1^, ^2^, ^3^, ^4^, ^18^, ^19^, ^20^, ^21^ ^]^ etc., as shown in Figure S1. Normalizing the obtained resistances by their mean value makes the fluctuation of the local resistance directly visible.

To measure distances beyond 250 µm, the electron conduction pathways must be considered. The supplement contains a detailed analysis of the electron conduction pathway. The probe's measuring field in physical contact with the specimen implies that every trace is in direct contact with it. An applied current on the outer traces injects electrons into the specimen, passing through until an inner trace is reached. For distances >250 µm, not all traces are used for monitoring but remain in contact with the specimen. Depending on the specimen's resistivity, electrons may favor passing through additional traces or solely through the specimen. The resistivity of the trace material (Cu) should be on the order of 10^−8^ Ω m, whereas for catalyst layers or GDL materials, it is in the order of 10^−3^ Ω m^[^ ^11^, ^23^, ^24^ ^]^ or 10^−5^ Ω m,^[^ ^29^ ^]^ respectively. Therefore, we concluded that for comparably high ohmic specimens, such as catalyst layers, the electrons will pass through the additional trace instead of the specimen. Overestimation of the specimen resistance by ignoring the contribution of the trace width will result in an error that is orders of magnitude smaller than the measured resistance. Thus, this effect can be neglected for specimens with resistivities of 10⁻^6^ Ω m or higher. The mathematical analysis and derivation of an equiv. circuit describing the electron paths and their effect on the measurement can be found in Figure S2 (Supporting Information).

## 3 Results & Discussion

### 3.1 Probe Geometry

**Figure 3:**
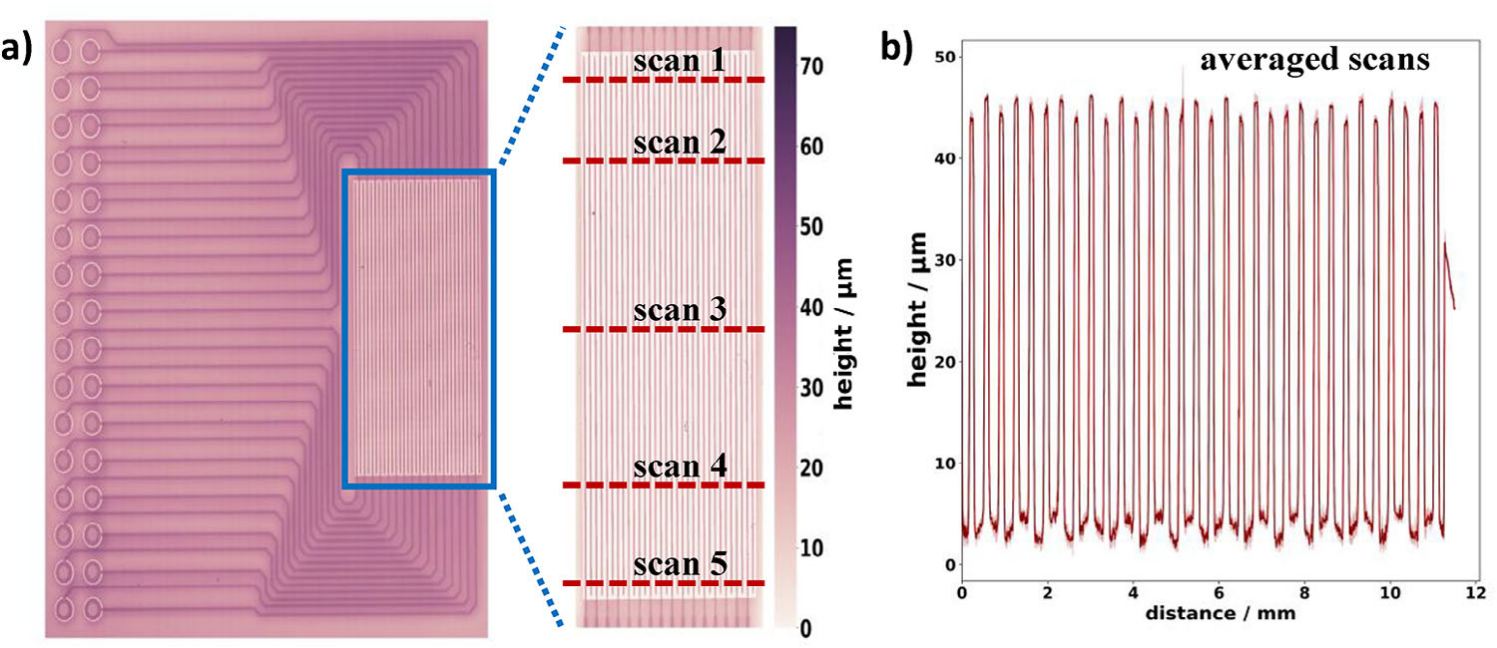
Analysis of the probe surface height distribution by non‐contact profilometry: a) Representing the entire probe surface with an enlarged view of the measuring field. Figure 3b shows several line profiles, scanned orthogonal to the traces as represented by the reddish dashed lines in (a). The 95% confidence interval in Figure 3b is indicated by the shaded area, calculated from five line scans. The line profiles recorded perpendicular to the traces of the measuring field exhibited an alternating height distribution. This emerged from the low height of the 250 µm distance between the traces and the actual height of the 100 µm width traces. The average height difference was 41.4 µm, which fits well to the height specification, mainly the final thickness of the copper (35 µm), with the additional nickel–gold surface finish (4–7 µm). However, the solder resist at the measuring field border (most right trace in Figure 3a) showed only a mean height of 27.5 ± 1.0 µm. This indicates that the traces protruded ≈14 µm with respect to the direct neighborhood, whereas they were 58 µm below the maximum height of the overall probe. Thus, proper contacting of the specimen with the measurement field must be considered. Especially for the smallest trace distance (250 µm), these height variations deteriorate the measurement, resulting in a non‐linear behavior of the potential scan response, violating Ohm's law. To adjust for these height differences and resulting unevenness, a (462 ± 4) µm‐thick silicon sheet was introduced between the specimen and stamp, ensuring proper contact between the specimen and measuring field, leading to *R^2^* values of > 0.99. The elasticity of the silicon sheet improves the pressure distribution and compensates for certain margins of error as they emerge from the stamp or probe.

The probe surface was studied by scanning the probe's surface with a non‐contact profilometer to see the impact of the solder resist coating on the height distribution. The obtained data presented in **Figure** **3**a shows a scan of the entire probe and a focused scan on the measuring field. The insulated traces covered with the solder resist protrude from the surface, with their maximum height ranging between 65 and 72 µm. To increase the clarity of the text, we will refer to these insulated lines as power lines in the following. The solder resist coated on the blank rigid board leads to height levels of ≈40 to 50 µm, which is slightly higher than the forgoing mentioned specifications. An explanation for the increased height could be that the power lines are very close to each other, increasing the thickness more strongly than expected. Analyzing the most crucial zone, the measuring field, shows that this zone exhibits a lower maximum height than the remaining probe. This can also be quantified by the averaged line profiles shown in Figure 3b.

### 3.2 Compression

The contact force was studied using pressure‐sensitive films at different compression forces (Figure S3, Supporting Information). The mean pressure applied to the measurement field was 0.35 MPa at an adjusted compression force of 0.50 kN (Figure S3a, Supporting Information). Increasing the compression force led to a linear increase of the distributed pressure for the compression forces >2.25 kN, with a qualitative analysis showing no visual differences regarding the pressure distribution (Figure S3d, Supporting Information). All further experiments were carried out at a compression force of 1.00 kN and 2.25 kN, which correlates with a mean pressure of 0.78 MPa and 1.94 MPa, respectively. Analyzing the pressure distribution indicated that the compression force of 1.00 kN (Figure S3b) is still insufficient to ensure proper electrical contact between the specimen and measuring field. We will show in the following how such an insufficient compression will influence the measured resistances. We also suggest using the probe's possibility to map the resistance at certain compression forces to find the most suitable compression force for each specimen that minimizes errors and fluctuations of the local resistance.

### 3.3 Probe Validation

**Figure 4:**
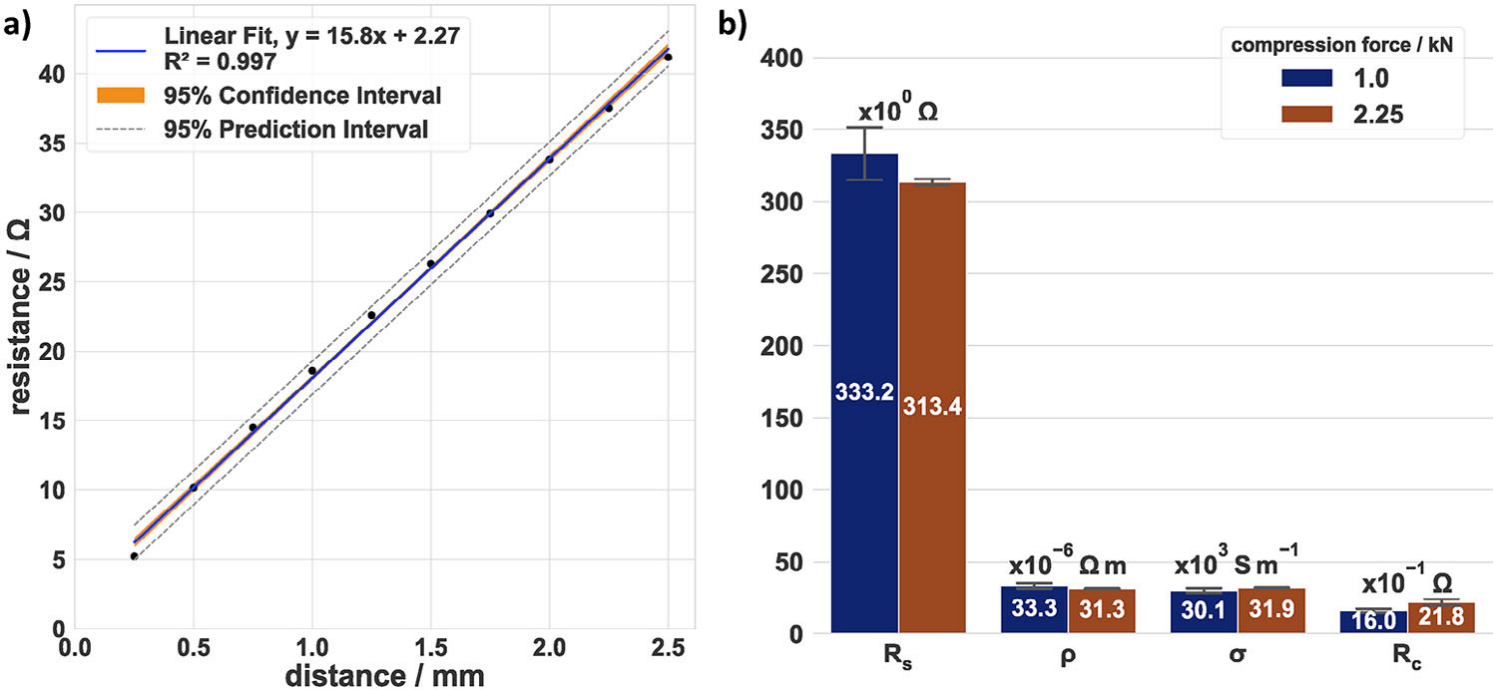
Validation results of the probe with an ITO–PET reference. The linear regression analysis in (a) shows a typical measurement of the sheet resistance for one specimen (2.25 kN, 25 rh%, 25 °C), whereas (b) shows the overall results for sheet resistance (R_s_), in‐plane electrical resistivity (ρ), in‐plane electrical conductivity (σ), and contact resistance (R_c_) for two different compression forces (25 rh%, 25 °C). This dataset included the use of four different probes, multiple measurements using one probe, and two compressions in the dataset. ρ and σ were calculated using the specified thickness of 100 nm. In (b), error bars represent the mean ± standard deviation (SD), sample size (n) = 5.

The described method was validated with a commercially available reference, namely a 100 nm‐thick ITO layer, coated on a 180 µm‐thick PET sheet and with a specified sheet resistance of 300 Ohm sq^−1^. The dataset presented in **Figure** **4**a shows the determined resistance as a function of the distance at a compression force of 2.25 kN. The 95% confidence interval is shaded in orange, and most of the data is scattered within these limits. The coefficient of determination (*R^2^*) was always >0.99 for all performed potential scan measurements at all distances measured. The standard error of the regression slope was 0.43 Ω mm^−1^ at 1.00 kN and 0.37 Ω mm^−1^ at a compression force of 2.25 kN. The resulting sheet resistance, together with the calculated in‐plane resistivity and conductivity, can be found in Figure 4b for 1.00 and 2.25 kN. At a compression force of 1.00 kN and 2.25 kN, the averaged ITO–PET sheet resistances were (333.2 ± 25.7) Ω and (313.4 ± 5.4) Ω, which differs from the reported resistance of the reference by less than 12% and 7%, respectively. The calculated in‐plane electrical resistivity at 1.00 kN was (33.3 ± 0.6) µΩ m, whereas the in‐plane conductivity was (30.1 ± 0.6) kS m⁻^1^. These values were in a typical range for the material, while it is assumed that the thickness was uniform throughout the sheet. However, when the thickness of porous electrodes is non‐uniform, both parameters are prone to error. The contact resistance (Figure 4b) was (1.6 ± 0.2) Ω at 1.00 kN and increased to (2.2 ± 0.6) Ω at 2.25 kN.

The deviation is in the range of the respective error. However, the absolute error decreased by half as the compression force increased. As a result, the relative SD decreased from 34% to 22% and the standard error decreased from 0.67 Ω to 0.62 Ω with respect to the intercept. However, convolving and interpreting the contact resistance remains challenging and error‐prone. Instead, spatial analysis of the specimen could further narrow this gap and improve understanding of the contact between the specimen and measuring field. Two distances of the probe were used for the 1D mapping, which were d = 250 µm and d = 2250 µm, with 29 and five equidistant configurations, respectively.

**Figure 5:**
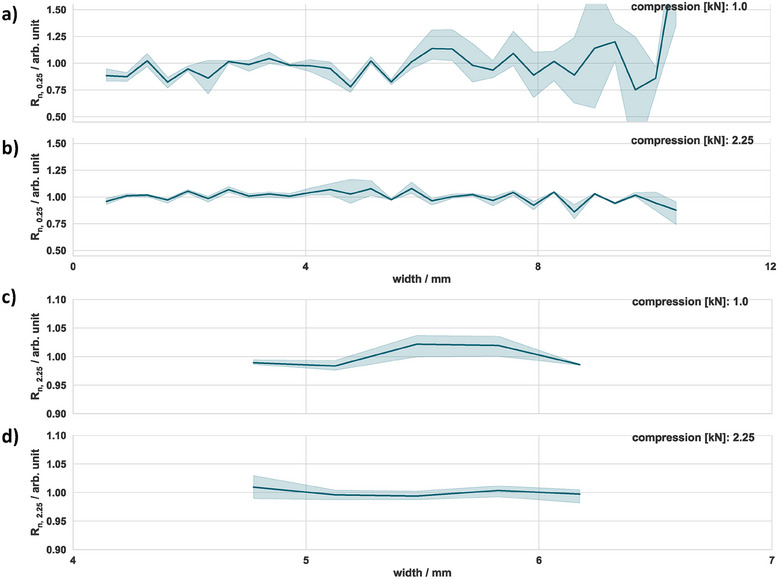
Mapping results of the reference specimen at two compression forces and distances a,b) 250 µm and c,d) 2250 µm. R_n_ is the resistance normalized by the average resistance measured for each distance. The shaded area represents the 95% confidence interval calculated from five samples.

The 1D mapping, presented in **Figure** **5**, was used to gain more insights regarding the homogeneity of the electrical contact between the specimen and measuring field. The x‐scale represents the location on the specimen, starting from the right end of the measurement field and progressing toward the center of the probe (refer to Figure 3a). The x‐coordinate represents the midpoint between the traces used for the measurement. The values on the y‐axis represent the deviation between the mean and measured resistance at the specified distance, whereas the shaded area indicates the SD. Figure 5 presents 1D mapping for the distances 250 (Figure 5a,b) and 2250 µm (Figure 5c,d) at two different compression forces (1.00 kN, 2.25 kN).

The resistance showed a higher SD as it approached the opposite side of the probe. This behavior can be explained by the protruding height and the number of power lines present in this region, which lead to a non‐uniform contact area between the measurement traces and specimen. Increasing the compression force to 2.25 kN reduced this effect remarkably, as can be seen in Figure 5a. The SD of the measured resistance decreased from 28% to 6% and, in turn, the variation around the median along the width of the measurement field decreased significantly. This was due to a more uniform contact area, ensured by the compression force and silicon sheet, which reduced misalignment between specimen and probe. As can be seen in Figure 5b, the relative SD was remarkably lower for a distance of 2250 µm, while it decreased from 2% to 1% with increased compression force. From this, we can conclude that the more locally we measure, the more error‐prone the measurement becomes, which could be due to local inhomogeneities of the probe, specimen or contact issues between both.

Based on our experience, we can offer the following advice when using this method. It is generally recommended to start measurements with larger distances (> 1500 µm) to ensure that the results are free from noise or other interfering effects. The effect of the compression force can then be examined. By monitoring the SD and coefficient of determination (R^2^), the optimum compressive force can be identified, thereby minimizing measurement errors. If the specimen of interest is coated on a substrate, the nature of the substrate must also be considered. It is strongly recommended that the compressive force be thoroughly investigated. The sample should be handled carefully to avoid damage or inaccuracy and should always be of the same size. For certain specimens, an extended period of preconditioning under measurement conditions may be beneficial to improve the reliability of the results.

After properly contacting the specimen, ensuring linearity, it is possible using the presented method to measure the local resistance fluctuation. However, the mechanical behavior during compression or local inhomogeneities of the specimen must always be considered.

We were able to determine the sheet resistance of the commercial ITO–PET reference with less than 7% error at a compression force of 2.25 kN, which proved that the proposed method worked and is an option to be utilized for other materials. For future probe designs, the power lines pathway should be rearranged and be more spatially separated from the measuring field.

While the nominal thickness of the commercial sample was known, the thickness of real samples needs to be determined experimentally. In the following we present the thickness determination for the utilized PTLs and GDLs, as well as the results from the optical approach for the determination of average catalyst layer thicknesses as described in the Experimental Section.

### 3.4 Thickness Determination

The thicknesses of the GDL and PTL were determined by measuring at least three specimens five times with a stationary thickness‐measuring device. The paper‐type GDL from Toray, TGP‐H‐120, possessed a mean thickness of (360 ± 4) µm. The thickness obtained from the microporous side of the SGL22BB was (197 ± 2) µm, whereas it was (194 ± 2) µm in the case of the woven HC2315. The titanium‐based PTL possessed a thickness of (358 ± 6) µm. All specimens showed a relative standard deviation (RSD) below 2%.

**Figure 6:**
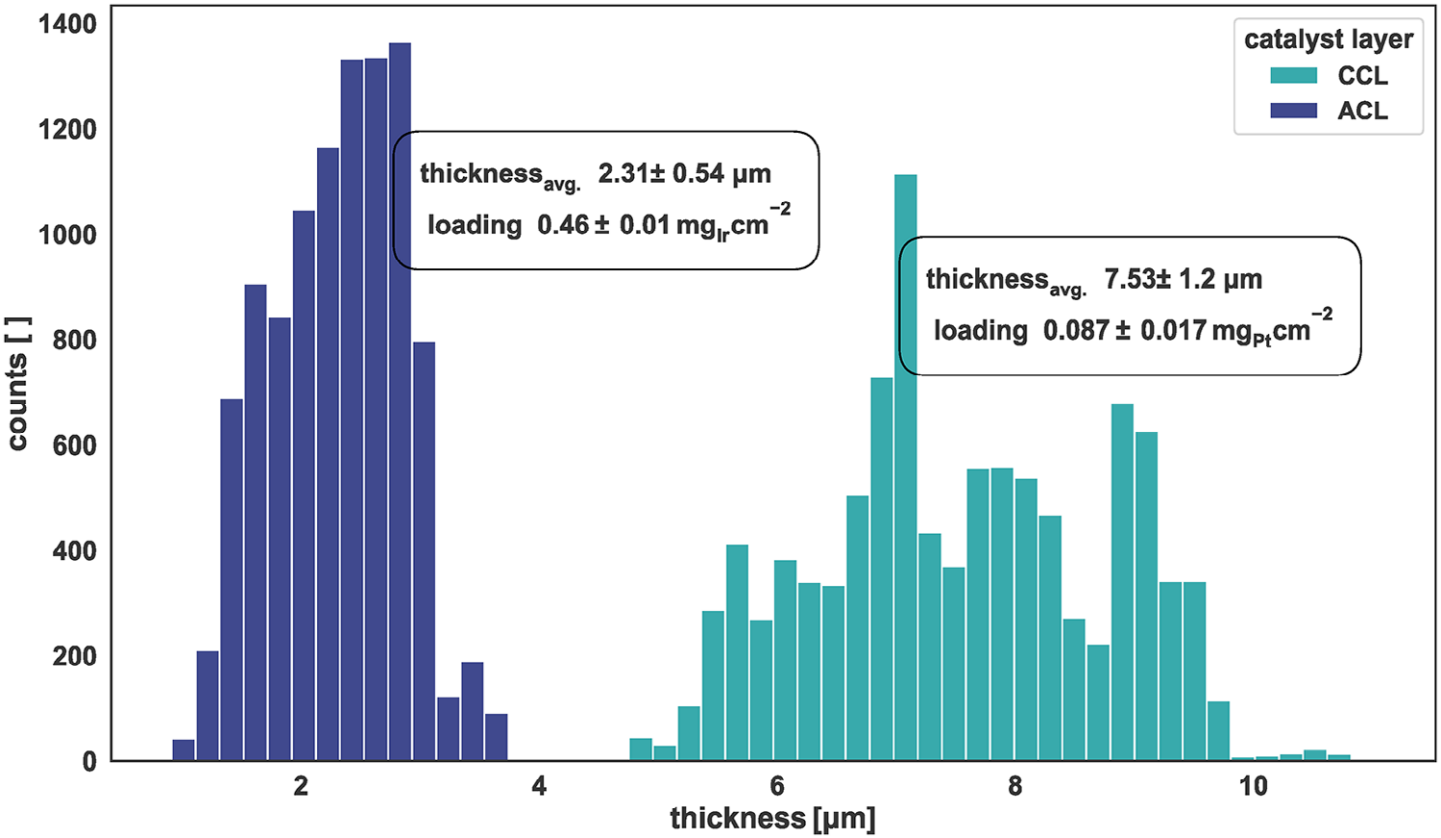
Obtained thickness distribution for catalyst layers by cross‐sectional analysis of AsB images. CCL denotes a PEM water electrolyzer cathode characterized by low metal loading but a high carbon fraction. The ACL represents optimized anode loading for the PEM water electrolyzer.

Image processing was used to obtain the thickness distribution from the catalyst layers, as presented in **Figures** **6** and S4.

The thickness of the anodic catalyst layer (ACL) was (2.3 ± 0.54) µm with 23% RSD while possessing a metal loading nearly five time higher than the CCL. The platinum‐loaded CCL possessed an average thickness of (7.5 ± 1.2) µm and a comparably lower RSD (16%).

In the case of the ACL, the minimum thickness was 0.91 µm, and the maximum was 3.71 µm, whereas the 25% percentile was at 1.89 µm and the 75% percentile at 2.73 µm. Instead, the CCL possessed a broader distribution with a minimum and maximum thickness of 4.73 µm and 11.0 µm, respectively. The 25% percentile was at 6.71 µm and the 75% percentile was at 8.47 µm. The distribution and related statistics visualized that the catalyst layers are films with a non‐uniform thickness distribution. This must be considered when determining the in‐plane electrical resistance of catalyst layers and calls for robust, empirical methods to overcome the limitations of the existing measurement methods.

Based on the determined specimen thicknesses, their resistances were determined with the new probe and the impact of the compression force for the different specimens was characterized.

### 3.5 Resistance and Resistivity of PEM Water Electrolyzer Components

#### 3.5.1 GDL and PTL Materials

**Figure 7:**
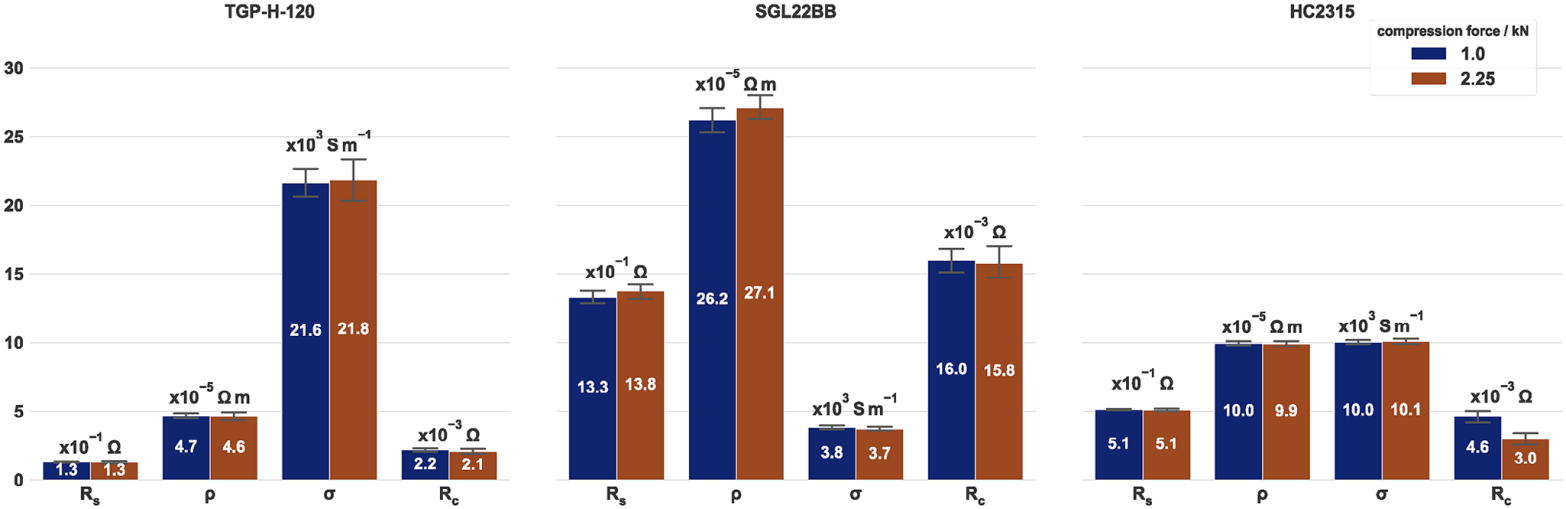
Determination of the sheet resistance (R_s_), in‐plane electrical resistivity (ρ), in‐plane electrical conductivity (σ), and contact resistance (R_c_) for typical carbon‐based GDL materials from three different suppliers. The error bars represent the mean ± SD, n = 3.

The comprehensive technical specification of GDL materials demonstrates that these materials are well‐studied. Thus, to extend the validation of the probe, three GDL materials were applied to determine the electrical in‐plane resistivity. The results presented in **Figure** **7** were compared to the technical specifications listed in **Table** **1**. Each GDL material has its own set of parameters in Figure 7, but a common y‐axis to facilitate comparison. For all specimens in Figure 7 the standard error of the slope was below 5 mΩ mm⁻^1^.

**Table 1:** Summary of the data gathered from the supplier specification available. In the case of the Freudenberg HC2315, the data of the complementary H23 was used and the specific resistivity was calculated by taking the presented thickness into account (denoted with *).

Material	Supplier	Thickness [µm]	In‐plane electrical resistivity [x10⁻^5^ Ohm m]
TGP‐H‐120	Toray Industries	370	4.70
HC2315	Freudenberg	170	13.6*
SGL22BB	SGL Carbon	215	33.0
2GDL10‐0.35	Bekaert	350	‐

Linear regression led to *R^2^* values >0.99, except for the TGP‐H‐120, which possessed an R^2^ >0.97 and *R^2^* >0.94 at a compression force of 1.00 kN and 2.25 kN, respectively. One explanation could be that the application of mechanical load changes the thickness, and an increased load affects the porosity and tortuosity of the GDL, ultimately deteriorating the fiber structure and reducing thickness.^[^ ^42^ ^]^ Applying a lower compression force or thicker silicon sheet reduces this effect. On the other hand, TGP‐H‐120 possessed the lowest in‐plane resistivity and deviation from the reference value provided by the technical specification. The highest in‐plane electrical resistivity was measured for SGL22BB, which furthermore deviated 18% from the provided value. The largest difference between the determined and specified value occurred for HC2315 (36%). However, the technical specifications were limited in providing information regarding the method and conditions used. Nothing was specified in the case of the TGP‐H‐120. The in‐plane resistivity of SGL22BB was determined by applying the van‐der‐Pauw method, and an internal standard procedure was utilized for HC2315. The difference could also have originated from differences in the thickness, as the compression load affects the thickness of the GDL materials and, therefore, the in‐plane electrical resistivity.^[^ ^29^, ^43^ ^]^ The contact resistance between the specimen and probe ranged between 2.1 mΩ and 16 mΩ among the materials tested.

The resistance determination of the titanium PTL had to be adjusted because of its comparably high electrical conductivity. This was caused by the potentiostat's constrained measurement window at low potentials (< 40 µV) and the probe's final copper trace thickness, which capped the maximum current at ≈400 mA. Therefore, instead of a linear potential ramp, a current ramp between 300 and 400 mA was applied to keep the potential as high as possible (Figure S5a). Due to the resulting smaller measurement window, the sheet resistance presented in Figure S5 (Supporting Information) scattered the most, although, as expected, it was also the lowest determined within this study. The sheet resistance of (3.8 ± 1.4) mΩ was thirty times smaller than that of the GDL materials, whereas the in‐plane electrical resistivity was in the typical dimension of metals (10⁻^6^ Ω m). However, *R^2^* of the regression lines was very low in some cases (< 0.34), which clearly indicates the current limitations of the method regarding highly conductive specimens. Using increased distances or thicker traces could improve the result from the probe's perspective, allowing a broader operation window. On the other hand, electrical testing must be adapted to reduce the noise within the measurement. In general, accessing the in‐plane resistivity of metallic PTLs with a more accurate method is still of great interest to study the crucial interface between the PTL or coated PTL and catalyst layer. From a technological point of view, the PTL's resistivity is not the limiting component in an electrolyzer.

One limitation emerges from the current probe design, which is not yet suitable for metallic PTLs. The low resistance of metallic PTLs, combined with the limitations of the current voltage and current window, results in large measurement errors. To overcome this challenge, the probe geometry must be specifically optimized for accurate measurements of metallic PTLs; however, maybe this design is then not suitable to measure other, e.g., GDLs or porous electrodes.

Another area for improvement of the presented measurement concept is the required measurement time, which remains high if all configurations are to be measured. This could be significantly reduced by automating the measurement process, which would then also allow high throughput testing.

#### 3.5.2 Catalyst Layers

**Figure 8:**
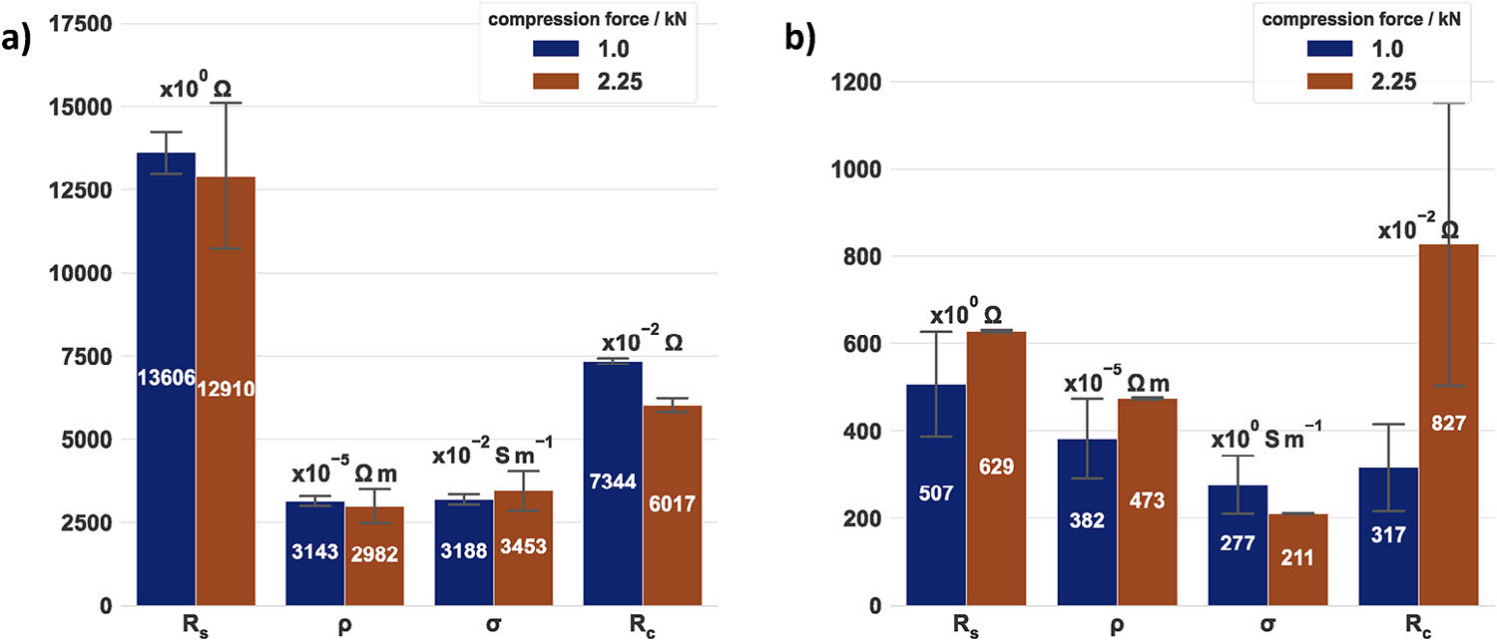
Comparison of the sheet resistance (R_s_), in‐plane electrical resistivity (ρ), in‐plane electrical conductivity (σ), and contact resistance (R_c_) for (a) an Ir‐based ACL (0.46 mg_Ir_ cm⁻^2^, average thickness of 2.31 ± 0.54 µm) and (b) a Pt‐based CCL (0.087 mg_Pt_ cm⁻^2^, average thickness of 7.53 ± 1.2 µm), each for two different compression forces. Error bars represent mean ± SD, n = 3.

This method aimed to characterize the resistance of catalyst layers coated onto membranes for PEM water electrolysis. Therefore, two different catalyst layers, representing a typical system for the anode (ACL) or cathode (CCL), are compared in **Figure** **8**. The ACL (Figure 8a) possessed a sheet resistance of (13.6 ± 0.9) kΩ at a compression force of 1.00 kN, which slightly decreased to (12.9 ± 3) kΩ after increasing the compression force to 2.25 kN. However, the RSD rose from 6% to 23% with increased compression force, which could indicate that the catalyst layer deforms with increased compression force, deteriorating the in‐plane connectivity between the particles. The inverse was the case for the CCL (Figure 8b), which showed a remarkably lower deviation of the sheet resistance of ± 4 Ω at a 2.25 kN compression compared to ± 170 Ω at 1 kN. In both cases, *R^2^* was > 0.99, whereas the standard error was below 9 Ω mm^−1^ for the ACL and smaller than 1 Ω mm^1^ for the CCL. The in‐plane electrical resistivity (*ρ*) was calculated from the average catalyst layer thickness for the ACL and the CCL. Both catalyst layers obtained an in‐plane electrical resistivity similar to values presented in the literature.^[^ ^11^, ^23^ ^]^ However, considering the 25% and 75% percentile of the thickness distribution for both catalyst layers, ρ could vary by up to ±18%. For the CCL, the resistivity calculated with the 25% percentile would be 11% less than that calculated with the average thickness. In the case of the 75% percentile, ρ would be 13% greater. The uncertainty of the thickness has a significant impact on the in‐plane resistivity. Therefore, it is recommended that the sheet resistance be used as a descriptor for catalyst layers in place of the in‐plane electrical resistivity.

The values of the contact resistance R_c_ are a factor ten lower for the CCL than for the ACL. However, it is only possible to represent this as a comparison between these two specimens, as the same probe and cables, etc. were used. This emphasizes the urgent need to tailor the catalyst layer–PTL interface and to develop micro‐PTLs, especially for the anode.

**Figure 9:**
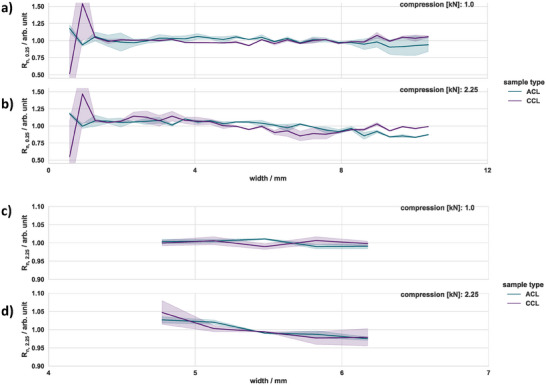
Result of the spatially‐resolved resistance for ACL and CCL at two compression forces with measurement distances of a,b) 250 µm and c,d) 2250 µm. The shaded area represents the 95% confidence interval that was calculated based on three samples.

**Figure** **9** presents the spatial mapping for both catalyst layers. At an adjusted compression force of 2.25 kN and a distance of 250 µm, the ACL had 25% and 75% quantile values ranging ≈0.89 and 1.08, respectively, whereas the CCL had corresponding values of ≈0.95 and 1.05. The differences in deviation could be attributed to the compression force, as it may alter the morphology of the catalyst layer.

As the distance increased, the resistance deviation for both catalyst layers decreased, with 25% and 75% quantile values ranging ≈0.98 and 1.02. To some extent, these trends are in line with the observations on the model ITO‐PET reference, i.e., that an increased distance leads to lower deviation in resistance. On the other hand, compression seems to have less influence on the resistance fluctuation compared to the reference, most likely because the PET‐support will have different mechanical properties than the membrane. Moreover, the catalyst layers are significantly thicker than the reference coating, i.e., several µm in comparison to a 100 nm ITO coating. Thus, the fluctuation visible in Figure 9 could be caused by deviations in thickness, loading displacements or different local ionomer concentrations. A more in‐depth analysis about such influences would require different conceptualized catalyst layers with variations in their composition and production.

Another point of great interest are the operating conditions. For the initial tests, conditions were set at 25 °C and 25% relative humidity to minimize any influence from the properties of the specimen. It is well known that the Nafion membrane swells with increased humidity, which disrupts the conduction network and leads to higher sheet resistance. To evaluate the effect of operating conditions, it is necessary to first analyze their effect on the specimen. The thickness of the catalyst layer could vary depending on the ionomer content, making it challenging to determine the thickness of the layer under wet conditions. However, the proposed method of determining the sheet resistance does not require knowledge of the thickness of the catalyst layer. This approach makes it possible to investigate the resistance of the catalyst layer under realistic operating conditions in future studies.

Our study identifies the anode catalyst layer as the component with the highest in‐plane resistivity in PEM water electrolyzers, emphasizing that improving the PTL‐catalyst contact through PTL design is more effective in improving performance than focusing solely on reducing the in‐plane resistivity of the catalyst layer.

However, a critical but often overlooked factor is the intrinsic relationship between electrical and thermal resistivity. This highlights the role of in‐plane resistivity in ensuring component durability and extending system life. Inefficient heat dissipation at the catalyst layer/PTL interface, particularly in areas of poor contact or local non‐uniformity within the catalyst layer, can create hot spots that accelerate degradation.

By applying the framework presented, we aim to improve the understanding of the interplay between the catalyst layer, interfacial contact, and system behavior. This work provides valuable insights to guide the design of PEM electrolyzers with improved performance and durability.

## 4 Conclusions

In this study, we presented and validated an empirical method for determining the sheet resistance of porous electrodes, such as GDL materials or catalysts. The sheet resistance could be determined with an error smaller than 7% for ITO–PET as a commercial reference. The methods were also validated for various GDL materials with different thicknesses, as well as catalyst layers, showing the broad applicability of the developed tool. Comprising 32 measurement traces in total, a spatial resolution of the local sheet resistance was also possible. This was demonstrated by characterizing the impact of the compression force on the electrical contact between the specimen and the measuring field.

To reliably characterize specimens with complex surface morphologies, such as catalyst layers with spatially‐varying thicknesses, we employed the open source‐based image processing of SEM images to determine the thickness distribution of the studied catalyst layers and obtain an average layer thickness. This was utilized as a basis to calculate the in‐plane electrical resistivity for both catalyst layers, which was discussed and compared to the sheet resistance. From our perspective, the sheet resistance offers a more reliable engineering parameter, as it is not dependent on the thickness and its non‐uniformity.

Comparing a Pt‐based catalyst layer with an Ir‐based one, we show that the Pt has ≈20‐times lower in‐plane electrical resistivity than the Ir‐based catalyst layer. Furthermore, 1D mapping revealed that in both cases the catalyst layer resistance was not dependent on the compression force, indicating intimate contact with the measuring field.

Overall, the presented measurement tool, based on advanced PCB technology, together with the applied methods, offers a versatile platform for the reliable determination of the sheet resistances of various components used in electrochemical reactors. Furthermore, the tool has the prospect of being further adapted for the study of the sheet resistance of all types of porous electrodes. An improved probe design could also consider the impact of power lines on the measuring field.

The proposed method, as an alternative to conventional four‐point or van der Pauw analysis, will help advance the understanding of important parameters for fuel cell and electrolyzer studies in a more detailed and, above all, comparable way between different laboratories. Therefore, the probe design will be made available as an open‐source technology with further possible extensions in the future.

## 5 Experimental Section

### References

For validation of the method, a commercially available thin film of indium tin oxide (ITO) coated onto polyethylene terephthalate (PET) was employed. This well‐studied material is used for a variety of electronic use cases, such as probes, or as an anode of organic light‐emitting diodes (OLEDs).^[^
^44^, ^45^, ^46^
^]^ The ITO‐PET from Sigma‐Aldrich, with a specified sheet resistance of 300 Ohm, was purchased. The sheet possessed a total thickness of 178 µm, which is within the range of a Nafion N117 membrane. On the other hand, the ITO coating was only 100 nm thick, which is one order of magnitude thinner compared to the thickness of typical catalyst layers.

### GDL and PTL Materials

Several carbon GDL materials (TGP‐H‐120, HC2315, SGL22BB) were used to perform further validation of the probe. The electrical in‐plane resistivity obtained from the technical specifications of the supplier is presented in Table 1, together with the respective thickness of the GDL. However, the specification regarding the testing procedure is limited. Furthermore, the study was extended by characterizing titanium–felt, which is typically used as the anodic PTL (2GDL10‐0.35).

### Catalyst Layer Fabrication

Anodic and cathodic catalyst layers were formed by spraying directly onto the membrane (N117, Chemours) using a benchtop ultrasonic coating system (ExactaCoat, Sono‐Tek). The ACL used iridium oxide (Premion, Alfa Aesar) with 0.46 ± 0.01 mg_Ir_ cm⁻^2^ as the catalyst. The cathodic catalyst layer (CCL) possessed a loading of only 0.087 ± 0.018 mg_Pt_ cm⁻^2^ using 20 wt.% platinum on carbon (HISPEC3000, Johnson Matthey). Both dispersions consist of a mixture of n‐propanol (Merck), deionized water (Milli‐Q, Merck Millipore), and were iced for 5 min before adding Nafion. Both catalyst layers possessed a final Nafion content of 11 wt.% but used different Nafion dispersions. A water‐based Nafion dispersion (D1021, Ion Power) was used for the ACL, whereas the cathodic catalyst layer used an isopropanol‐based Nafion dispersion (LQ‐1115, Ion Power). After adding the Nafion, the ice bath‐cooled dispersion was homogenized by means of an ultrasonic horn for 30 min. The catalyst loading was determined by weighing them with a balance (AG204, Mettler Toledo) after spraying them onto borosilicate glass.

### Thickness of the Materials

To calculate the in‐plane electrical resistivity, knowledge regarding the specimen thickness is necessary. In the case of the GDL and PTL materials, the thickness was obtained using a stationary thickness measurement device (DM 2010, Wolf Messtechnik GmbH). On the other hand, the catalyst layer thickness was evaluated using a cross‐sectional analysis and scanning electron microscopy (SEM) using a Zeiss Gemini Ultra Plus. However, the thickness is usually determined manually without any standard procedure, which gives no insight into the distribution. Therefore, in the following, a simple method for applying image processing to angle‐selective backscattered electron (AsB) images to obtain the thickness distribution of the catalyst layers, is described.

### Thickness Determination from Cross‐Sections

**Figure 10:**
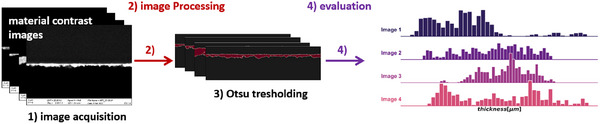
Determination of the catalyst layer thickness from cross‐sections. The used material contrast images are processed by Python‐based open‐source tools.

The proposed method is similar to commonly applied approaches for obtaining the particle size distribution from microscopy images, but instead, it determines the catalyst layer thickness.^[^ ^47^, ^48^, ^49^ ^]^ The principle utilized to gain the thickness distribution from the cross‐sectional AsB images is schematically shown in **Figure** **10**. The material contrast images used are processed by Python‐based open‐source tools. This study employed scikit‐image, an open‐source image processing library available for the Python environment. Analyzing cross‐sectional AsB images to obtain the thickness distribution of catalyst layers requires the conversion of pixels into a length unit. Thus, the pixels of the scale were counted to obtain their lengths‐per‐pixel. This value was then used to determine the thickness of the respective catalyst layer.

The cross‐sectional AsB images require binarization prior to evaluation. Several methods are proposed to set this threshold value, commonly to distinguish between the image background and foreground. In the case of the cross‐sectional analysis, this value must differentiate between the catalyst layer and membrane. AsB images have the advantage that their contrast depends on the difference in the atomic number of the materials depicted. Thus, the ACL made out of iridium oxide can be easily differentiated from the Nafion membrane using the Otsu algorithm to set the threshold for transforming the image into a binary one.^[^ ^50^ ^]^ In this binary image, only the catalyst layer possessed a pixel value of one.

However, a catalyst layer made of 80 wt.% carbon and 20 wt.% platinum was more difficult to distinguish from the Nafion membrane due to the high carbon content in both the membrane and catalyst layers. In this case, a multi‐Otsu algorithm,^[^ ^51^ ^]^ specifying several thresholds and choosing the best one by comparing different thresholds and their impact on the thickness determination, is used.

The average thickness for the presented catalyst layers was determined using ten different images at a magnification of 10 kx. The x‐direction of the image possesses a width of 1024 pixels. The accuracy of the obtained thickness distribution depends on the number of thickness determinations per image. Thus, each image provides a thickness distribution with 1 024 counts in total, which was then merged for all images taken from the same cross‐section. The thickness was determined at each of the 1 024 pixels in the x‐direction by summing the pixels in the y‐direction. These values were then converted into the thickness by the length‐per‐pixel value. Figure 10 illustrates the steps of the image processing method. More details for the individual samples are given in the Results and Discussion.

### Characterization of the Topology

The probe topology was studied using a non‐contact profilometer (CT‐300, cyberTECHNOLOGIES GmbH) with a lateral resolution of 0.05 µm and a step width of 5 µm.

### Compression Analysis

To measure the distribution of the force applied to the specimen, three different pressure‐measuring films (Prescale, Fuji Film CMV Hoven GmbH) covering a pressure range of 0.2 MPa to 10 MPa were used. It was expected that the adjusted compression force would not be equal to the force present on the measuring field and specimen. Thus, the pressure‐measuring films were placed on top of the measuring field instead of a specimen and everything was assembled as depicted in Figure 2c. The adjusted compression force displayed by the compression force sensor ranged from 0.5 to 5.0 kN.

### Statistical Analysis

All statistical calculations were performed using Python libraries such as SciPy, Pandas, NumPy, or Seaborn. For values reported with associated errors or when error bars are mentioned, the data is expressed as the mean ± SD). These values are based on a minimum of three experimental data sets to ensure reliability and accuracy.

## Conflict of Interest

The authors declare no conflict of interest.

## Supporting information



Supporting Information

## Data Availability

The data and technical drawings are provided by *Jülich* Data.https://data.fz‐juelich.de.
