# Supplementary material for: Innovative Method for Reliable Measurement of PEM Water Electrolyzer Component Resistances
Source: Small Methods. 2025 Jan 24;9(7):2401842. doi: 10.1002/smtd.202401842 (PMC12285620; doi:10.1002/smtd.202401842)
Supplement: Supplementary file 1 — Supporting Information [file SMTD-9-2401842-s001.docx]

Supporting Information

Innovative Method for Reliable Measurement of PEM Water Electrolyzer Component Resistances

Nikolai Utsch*, Florian Berg, Fabian Scheepers, Sebastian Holtwerth, Meital Shviro, Werner Lehnert, and Anna K. Mechler*

The Supplementary Information includes:

Figure S1. Overview of the configuration that can be utilized to determine resistance. The two outer traces (P+, P-) are used to apply the current/potential, whereas the inner traces monitor the response. The number in the gray shaded background is the sum of the total possible measurements per distance.

Paragraph 1 Mathematical analysis and derivation of an equivalent circuit.

Figure S2. Theoretical considerations (a) regarding the equivalent circuit of the probe contacted with the sample and (b) the analysis of the deviation emerging from the assumed distance traveled by the electrons. The framed schematics in (b) represent the two cases as a function of the ratio between the sample and trace resistance. The green bar of the scheme denotes the rigid board, the golden square represents the traces, and the brown bar the sample contacted to them.

Figure S3. Distribution of the compression onto the measuring field. The compression force was adjusted to (a) 0.50 kN, (b) 1.00 kN, (c) 2.25 kN, and (d) 5.00 kN.

Figure S4. Analysis of the cross-sections for the anode (a–c) and cathode catalyst layers (d–f). Image processing of the cropped raw cross-sections (a, d) led to the extracted binary image determining the thickness (b, e) and the equally weighted image composition to inspect the image processing quality (c, f).

Figure S5. The determined sheet resistance of the titanium-based PTL and in-plane electrical resistivity/conductivity lying in a range that is typical for metals.

**Figure S1**. Overview of the configuration that can be utilized to determine resistance. The two outer traces (P+, P-) were used to apply the current/potential, and the inner traces monitored the response. The number in the gray shaded background is the sum of the number of possible measurements per distance.

**Mathematical Analysis and Derivation of an Equivalent Circuit**

Measuring distances larger than 250 µm necessitates considerations regarding the electron conduction pathway. The measuring field of the probe contacted with the sample implies that every single trace of the measuring field is in physical contact with the sample. An applied current on the outer traces injects the electrons into the sample. The electrons pass solely through the sample until they reach an inner trace. In the case of a total measuring distance of 250 µm, this inner circuit is used to monitor the response. However, for distances > 250 µm, not all inner traces are used for monitoring but remain in contact with the sample. Depending on the resistivity of the sample, it is either more favorable for the electrons to pass through the additional trace than through the sample, or, if the sample resistivity is much lower than the resistivity of the trace, it is more likely to pass solely through the sample. The resistivity of the trace material (Cu) should be on the order of 10^-8^ Ω m, whereas the resistivity for catalyst layers or GDL materials is on the order of 10^-3^ Ω m or 10^-5^ Ω m, respectively. In the best case, the interface resistance of the copper trace and sample surface should be below 100 mΩ. It is likely that the electrons pass through the traces and the sample, especially if the sample and trace resistances are similar. To understand the two limiting cases more profoundly, we used a general mathematical expression (Equation S1) corresponding to an equivalent circuit (Figure S2a) representing the measurement.

$R=N\cdot\rho_{A,m}\cdot d_{m}+2R_{c}+R_{\mathrm{su}}+ \left( N-1 \right) \frac{\left( 2R_{c}+d_{t}\cdot\rho_{A, t} \right)\cdot d_{t}\cdot\rho_{A, m}}{2R_{c}+d_{t}\cdot\left( \rho_{m}+\rho_{A,t} \right)}$ Equation S1

The measured resistance (*R*) can be described as a serial function of the sample resistance (*R_m_*) and trace resistance (*R_t_*) and must be enhanced by the contact resistance (*R_c_*) between the sample and traces, as well as the resistances emerging from cables, solder joints, and others represented as (*R_su_*), shown in Figure S2a. Assuming a constant cross-sectional area (*A*), the sample resistance (*R_m_*) can be expressed as $R_{m} = {(\rho}_{m} / A)\cdot d_{m}$, where *d_m_* is the distance (*d_m_* = 250 µm) between the traces and $\rho_{A,m}$ the resistivity of the material normalized to the cross-sectional area. Similarly, the trace resistance (*R_t_*) is expressed as the trace material resistivity $(\rho_{A,t})$, with a constant cross-sectional area and trace width (*d_t_* = 100 µm).

If *R_m_* >> *R_t_* the main contribution to the measured resistance emerges from the material tested, allowing *R_t_* to be neglected. This consideration must be accounted for in the total length (*l*) assumed for the evaluation of distances *d_m_* > 250 µm, leading to two limiting cases. Equation S2 assumes that *R_m_* >> *R_t_* and therefore the total length (250–2500 µm) is equal to the number (N) of distances between the traces. Contrary to that, if *R_m_* << *R_t_* the total length (250–3450 µm) must take into account the additional widths of the traces (*d_t_* = 100 µm); see Equation S3. However, these assumptions neglect possible current crowding or non-uniform contact resistances.

$l=N\cdot d_{m}$ Equation S2

$l=N\cdot d_{m}+\left( N-1 \right)\cdot d_{t}$ Equation S3

From the equivalent circuit and Equation S1, it becomes clear that if the sum of contact resistances and trace resistances is far smaller than the sample resistance, case 1, described by Equation S2, occurs instead of case 2, as expressed by Equation S3. Thus, assuming case 2 for low conductive materials would lead to large errors regarding the determined resistance. A general analysis of the error is presented in Figure S2b for both cases. The deviation of the measured resistance from its theoretical value (*R_theo_*) of a given sample is scaled by the minimum and maximum on the y-axis, and on the x-axis, the ratio between *R_t_* and *R_m_* is presented. The greenish to blueish colored lines referred to in case 2 with the total length calculated by Equation S3, showing that the error increases if R_m_ >> R_t_, indicate that the electrons are not flowing solely through the sample but also pass through the traces. Thus, it is valid to assume case 1 and Equation S2 for determining the resistance of low conductive materials, as can also be seen by the line represented by a magenta color code. On the other hand, it is not appropriate to assume case 1 for highly conductive samples, but case 2 would be. For case 1, the maximum error reaches a threshold of around 0.36 if *R_m_* << *R_t_*. Case 2 results in a maximum error at a threshold of around 0.26 for *R_m_* >> *R_t_*. The threshold can be explained by the geometric ratio (*r*) between d_m_ and d_t_ converging towards a limit at approximately 0.71. The theoretical resistance used in Figure S2b originated from the reference material and was later used for validating the method. However, the calculation in Figure S2b assumed that there is no additional contact resistance between the current supply (*R_i_*) and the sample and that no resistance emerged from the set-up.

**Figure S2**. Theoretical considerations (a) regarding the equivalent circuit of the probe contacted with the sample and (b) the analysis of the deviation emerging from the assumed distance traveled by the electrons. The framed schematics in (b) represent the two cases as a function of the ratio between sample and trace resistance. The green bar of the scheme denotes the rigid board, whereas the golden square represents the traces and the brown bar the sample contacted to them.


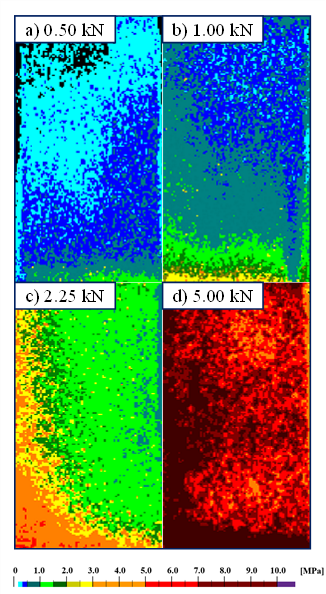


**Figure S3**. Distribution of the compression onto the measuring field. The compression force was adjusted to (a) 0.50 kN, (b) 1.00 kN, (c) 2.25 kN, and (d) 5.00 kN.

**Figure S4**. Analysis of the cross-sections for the anode (a–c) and cathode catalyst layer (d–f). Image processing of the cropped raw cross-sections (a, d) leads to the extracted binary image determining the thickness (b, e) and the equally weighted image composition to inspect the image processing quality (c, f).

**Figure S5**. Determined sheet resistance of the titanium-based PTL and in-plane electrical resistivity/conductivity with typical values for metals.
